# The Impact of Regular Physical Activity on Children’s Health Parameters: A Critical Literature Analysis of Metabolism, the Musculoskeletal System, Respiratory System Diseases, and Neuropsychiatry

**DOI:** 10.7759/cureus.98198

**Published:** 2025-11-30

**Authors:** Mateusz Piszka, Maria Kubicka, Julia Zerdka, Patryk Brasse, Aleksandra Owczarska, Hubert Dacyl, Jakub Bartkowski, Jan Banach, Eliza Kwapien

**Affiliations:** 1 General Practice, Wojewódzki Szpital Specjalistyczny Nr 5 im. św. Barbary w Sosnowcu, Sosnowiec, POL; 2 General Practice, Samodzielny Publiczny Szpital Kliniczny im. Andrzeja Mielęckiego w Katowicach, Katowice, POL; 3 General Practice, Miejskie Zakłady Opieki Zdrowotnej w Żorach, Żory, POL; 4 Psychiatry, Szpital Powiatowy w Limanowej Imienia Miłosierdzia Bożego, Limanowa, POL

**Keywords:** general pediatric, lipid profiles, musculoskeletal health, neurodevelopment, obesity and diabetes, pediatric cardiologist, physical training

## Abstract

Physical activity is widely recognized as beneficial to children’s health, but knowledge about the positive effects depending on the intensity and regularity of physical exercise remains insufficient. This study aimed to compile research covering a broad pediatric population and identify the most significant, commonly observed benefits, as well as to analyze detailed research results concerning the most common health disorders, such as obesity, diabetes, and respiratory system diseases. It has been shown that the greater the regularity and intensity of exercise, the better the health parameters observed in children. The benefits of physical training also include improved subjective well-being, greater self-esteem, better emotional regulation, and overall improvement in cognitive function. They increase bone mineral content and mineral density, which leads to increased strength and stability of the skeleton. Habits formed during childhood have a significant impact on the potential development of diseases in adulthood; therefore, interdisciplinary cooperation between specialists is advisable, and, in certain cases, the preparation of individual exercise interventions, especially in the case of coexisting endocrine disorders.

## Introduction and background

Physical activity is fundamental to the musculoskeletal development, physical health, and mental well-being of the entire pediatric population. Numerous studies have confirmed that children with higher activity levels exhibit improved bone density, reduced obesity, and better overall physical fitness. Even small amounts of activity yield significant health benefits, particularly for high-risk groups such as children with obesity [1]. Specifically, moderate-to-vigorous physical activity (MVPA) has a positive influence on cardiometabolic risk factors, including high-density lipoprotein (HDL)-cholesterol (HDL-C) levels and blood pressure. Clinical evidence suggests that 30 minutes of daily MVPA can reduce waist circumference by up to 1.5 cm [2], highlighting the clear therapeutic potential of active lifestyles for the pediatric population. Establishing these habits during the developmental period is critical, as childhood lifestyle patterns often persist into adulthood, directly affecting the risk of chronic conditions such as insulin resistance and cardiovascular disease [3]. For overweight and obese children and adolescents, physical training has been shown to effectively reduce fasting glucose, fasting insulin, homeostatic model assessment for insulin resistance (HOMA-IR), and body weight. Unlike pharmacological interventions, which may carry side effects and are more frequently utilized by populations with excess weight, physical activity offers an accessible, cost-free, and safe therapeutic strategy. Given these extensive benefits, identifying effective methods to increase physical activity in overweight and obese children and adolescents is a priority [4]. Addressing the serious long-term consequences of physical inactivity requires early intervention to prevent adult-onset diseases. Consequently, mitigating these negative health effects demands an interdisciplinary approach where pediatricians, teachers, and parents collaborate to educate and activate children across all age groups [5].

## Review

Methodology

This narrative review is based on a comprehensive literature search conducted in the PubMed database, covering the period from 2010 to 2025. We employed a combination of keywords and MeSH terms related to physical activity, child development, and health outcomes, specifically “physical activity,” “motor development,” “cognitive development,” “psychosocial outcomes,” “children,” and “pediatric population.” The inclusion criteria were restricted to studies published in the English language that specifically concerned the pediatric population. To ensure the reliability of conclusions without the need for a new quantitative synthesis, we prioritized high-level evidence, particularly existing systematic reviews and meta-analyses, though in research areas with limited publications, the majority of available studies were considered to ensure adequate representation of the field. As this article is a narrative review, no formal risk-of-bias tools were applied; instead, the overall quality of evidence was assessed narratively by examining included studies for methodological clarity, sample characteristics, and relevance to the review’s scope. Across the literature, common strengths such as clear definitions of physical activity parameters were weighed against limitations such as small sample sizes and heterogeneous designs when interpreting findings. Regarding statistical methods, no new analyses were performed; all statistical values, including p-values and confidence intervals, presented in the text are citations from the primary sources used to illustrate the strength of the existing evidence rather than results of independent re-calculation.

Physical activity as a multilevel determinant of pediatric health

It is widely believed that all physical activity in children is beneficial to their development. However, after analyzing the research, a consistent link with health indicators was found mainly for moderate- and high-intensity physical activity [6]. In a group of pediatric patients aged three to six years, increased physical activity correlated with better bone health indicators and a lower obesity rate. The results of the impact on cognitive function were also compared, but no clear correlation was confirmed [3]. Subsequent studies have shown that high-intensity physical activity translates directly into metabolic health, as it improves body mass index (BMI) and lipid profile. Moderate physical activity is also valuable and is associated with better insulin resistance indicators [7]. Children with juvenile idiopathic arthritis (JIA) are a special group of pediatric patients. Physical activity programs have been used in this group and shown to be clinically effective in alleviating pain intensity, increasing functional capacity, and reducing disability. The overall health-related quality of life in this population is improving [8]. However, according to the results, there are certain risk groups of adolescent patients, such as obese individuals, for whom even a small amount of physical activity can bring health benefits [1]. According to the 2020 WHO guidelines, some physical activity is better than none, and more physical activity is better for optimal health outcomes. New recommendations on reducing sedentary lifestyles have also been added. These guidelines emphasize the importance of regular aerobic and muscle-strengthening exercise. For the first time, specific recommendations for certain social groups have been included. These include pregnant and postpartum women, as well as people with chronic diseases or disabilities. [9]. Physical activity is not just recommended for children. Studies based on an objective assessment of parents’ physical activity have shown a strong correlation between the physical activity of children and parents, and the strength of the correlation does not depend on the intensity of the activity measured, for example, by the number of steps [10]. The negative health effects of a lack of or insufficient activity in children can be serious and can translate into health problems in adulthood. It is important to promote physical activity in the pediatric population, both by pediatricians and teachers, from preschool teachers to physical education teachers. Many negative health consequences can be avoided, making interdisciplinary cooperation important [5].

Physical activity as a determinant of metabolic health based on an analysis of studies of patients with type 1 diabetes

In pediatric endocrinology, physical activity plays many important roles, especially in preventing overweight and obesity, which are themselves risk factors for further metabolic and hormonal disorders. Its role in a targeted treatment strategy for carbohydrate metabolism disorders and insulin resistance is also significant [4]. Physical activity increases insulin sensitivity and reduces the risk of metabolic syndrome, regardless of changes in BMI. Regular exercise, both aerobic and resistance training, contributes to beneficial changes in body composition, adipokine levels, and pancreatic hormone function. However, depending on age, gender, and type of exercise, the effectiveness of physical activity may vary. This points to the need for individualized exercise interventions in the prevention of endocrine disorders in children [11]. Most studies analyze the impact of exercise on the health parameters of children with diabetes. However, these patients also experience difficulties related to physical activity. Patients fear hypoglycemia, loss of glycemic control, and insufficient knowledge about managing physical exercise. These fears are well-founded. A 2017 study published in The Lancet showed that aerobic exercise is associated with a decrease in blood glucose levels, while anaerobic exercise may be associated with a temporary increase in glucose concentration. Unfortunately, both forms of exercise can cause delayed hypoglycemia during recovery [12]. An analysis of 55 studies involving more than 3,000 children and adolescents showed that physical exercise was associated with a reduction in fasting insulin and HOMA-IR. Importantly, however, these results improved with frequent and prolonged activity. The minimum required dose of exercise was approximately 900 to 1,200 MET-minutes per week [13]. Regular exercise can help patients achieve several goals: it improves the cardiovascular risk profile in pediatric patients and lowers HbA1c by approximately 0.3% in the pediatric population. Body composition, cardiorespiratory fitness, endothelial function, and blood lipid profile (i.e., triglycerides and total cholesterol) improve with regular activity in children and adolescents with type 1 diabetes [12]. Adipokines also play a role in the development of diabetes. Their impaired secretion contributes to the development of insulin resistance, atherosclerosis, and other complications with a latent onset. Physical exercise does not affect leptin and resistin levels, but has been shown to be associated with a significant increase in adiponectin concentration, which is reduced in diabetes [14].

Impact of physical activity on neuropsychiatric functioning in the pediatric population

Consistent physical activity interventions in healthy adolescents are associated with substantial improvements across several psychosocial domains. These benefits include enhanced subjective well-being, greater self-esteem, improved emotional regulation (including reduced anxiety and depressive mood), and superior emotional intelligence. Collectively, these psychological gains enhance self-regulatory capacities and intrinsic motivation, which directly support the adoption of healthier lifestyles and increase the individual’s perceived self-efficacy in maintaining long-term health [15]. Engagement in organized sports participation and physical activity inherently facilitates exposure to heterogeneous social cohorts, thereby quantitatively increasing social contacts and qualitatively deepening peer integration. Moreover, physical activity optimizes the behavioral and cognitive components underpinning social competence, which translates to a comprehensive enhancement of long-term health outcomes [16]. In line with findings reported by Yang et al., evidence gathered during the pandemic demonstrates a significant inverse correlation between daily physical activity and the prevalence of common mental health disorders in adolescents. Specifically, the study established that insufficient physical activity, defined as engaging in fewer than 30 minutes of physical activity per day, was independently associated with a markedly higher probability of developing both depressive and anxious symptomatology within this vulnerable population. Therefore, promoting adequate daily physical activity is critical for mental health protection [17]. Sustained physical activity and structured exercise programs utilized during the pandemic have been empirically linked to favorable outcomes, demonstrating an alleviating effect on symptoms of depression and anxiety [15]. Physical exertion induces beneficial neurotransmitter shifts, primarily marked by augmented activity within the serotonergic and dopaminergic pathways. As detailed in the reviewed literature, this biochemical upregulation is fundamentally important for stabilizing affective state and promoting an anxiolytic effect (reducing anxiety) [17]. Regarding specific developmental stages, Pacheco et al. reported that physical activity interventions are crucial for increasing neurocognitive outcomes in early childhood [18]. In contrast, for school-aged children and adolescents, the cognitive demands of physical activity become more complex. Successful participation in demanding activities requires these older children to utilize core executive functions, including robust attentional focus, efficient working memory, and consistent inhibitory control to filter external distractions. As physical exercise intrinsically relies on these same executive functions (inhibition, decision-making, and processing speed) to optimize performance, this functional requirement promotes a generalized improvement in overall cognitive function in this older demographic [19].

Physical activity and cardiovascular health in pediatric patients

Both cardiorespiratory conditioning and concurrent aerobic-resistance training were found to correlate positively with improvements across several cardiovascular disease risk indicators in pediatric and adolescent patients affected by obesity [20]. In the general pediatric and adolescent cohort, superior cardiorespiratory fitness correlated significantly with enhanced health outcomes across 17 distinct markers. This included beneficial improvements in adiposity measures (e.g., reduced skinfold thickness, smaller waist circumference, and lower BMI) and critical cardiometabolic and vascular health indicators (e.g., lower prevalence of metabolic syndrome, improved insulin sensitivity, fasting insulin and HOMA-IR, and a more favorable lipid panel, notably total cholesterol, triglycerides, HDL-C, and central pulse-wave velocity) [21]. Both endurance-based (aerobic) and strength-based (resistance) exercise regimens were effective strategies for treating cardiometabolic risk in overweight and obese school children. They correlated with significant reductions in adiposity (BMI and body fat percentage) and the atherogenic lipid panel (triglycerides, low-density lipoprotein (LDL), and total cholesterol) [22]. Kelley et al. documented that the observed improvements in the lipid profile were restricted to aerobic training. Their data showed that aerobic exercise was the sole intervention associated with a statistically significant elevation in HDL-C. Regarding LDL-cholesterol, statistically significant reductions were observed specifically in aerobic exercise groups (p = 0.001). Compared to the baseline mean, these changes corresponded to a relative decrease of 12.2%, indicating a clinically meaningful improvement in lipid profile [20]. Recent evidence indicates that engaging in exercise is associated with a clinically relevant reduction in blood pressure, typically ranging from 5 to 8 mmHg. Furthermore, non-pharmacological approaches, particularly those involving lifestyle modifications, have proven effective in achieving blood pressure reduction among healthy adolescents. Consequently, physical activity protocols are now established as a primary, key component in strategies aimed at lowering systemic blood pressure [23]. Myocardial remodeling in response to physical training is evident across the pediatric age range, extending even to pre-pubertal athletes. This cardiac adjustment predominantly manifests as changes in structural metrics (e.g., ventricle size or wall thickness). Among junior endurance athletes, a significant elevation in ejection fraction was documented [24].

Physical activity as a determinant of musculoskeletal health in the developmental age

Exogenous determinants, including nutritional status and engagement in physical activity, significantly influence the attainment of adult peak bone mass (PBM), contributing an estimated 20% to 40% of its variance. Consequently, regular physical activity is an established strategy for health promotion and chronic disease prevention across the lifespan. Regarding the musculoskeletal system, the skeleton exhibits a rapid and favorable biological response to mechanical loading, initiating prompt bone remodeling to enhance structural integrity [25]. Lifestyle factors, specifically nutritional intake and sufficient physical activity, represent key determinants of skeletal development. When managed optimally throughout childhood and adolescence, these elements are crucial for maximizing PBM. Continuing these healthy behaviors into maturity is essential for maintaining bone integrity and contributing to a robust adult skeleton [26]. Physical activity profoundly influences skeletal homeostasis via complex endocrine and biomechanical signaling. Exercise stimulates muscles and adipose tissue to release regulatory factors, notably myokines (such as irisin) and adipokines (such as leptin), which act on distant tissues to modulate bone cell function. Concurrently, physical strain promotes the essential mechanical loading of the skeleton through muscle contraction, while also initiating cellular processes such as the activation of brown adipose tissue and the regulation of autophagy in osteoblasts. These combined effects collectively enhance bone mineral content (BMC) and bone mineral density (BMD), leading to a demonstrable improvement in skeletal strength and stability [27]. Regular physical activity, delivered at an appropriate intensity and volume, can significantly enhance BMC and BMD during development, which is critical for attaining a higher PBM. This accrued skeletal strength acts as a protective factor, helping mitigate bone loss and reduce the risk of osteoporosis later in adult life [26]. Sustained high-level physical activity throughout childhood and adolescence is strongly correlated with beneficial skeletal adaptation, including increased PBM. Simultaneously, it supports superior neuromuscular efficiency and greater muscle strength. These developmental advantages ultimately lead to a markedly reduced incidence of fractures both during the growth period and later in adulthood [28]. To achieve optimal health benefits, pediatric patients between the ages of 6 and 17 require a minimum of 60 minutes daily of MVPA. This regimen should comprehensively integrate activities that promote cardiorespiratory fitness (aerobic exercise), enhance skeletal muscle strength, and stimulate bone integrity (bone-strengthening activities) [29].

Impact of physical activity interventions on the progression of pediatric lung disorders

Exercise in Pediatric Asthma

Gaining comprehensive insight into the influence of physical activity and BMI, both acknowledged as readily modifiable determinants, is crucial. Furthermore, integrating the systematic screening of exercise status as a clinical vital sign is essential in assessing the developmental trajectory of asthma in children. This understanding will facilitate the design of targeted prophylactic and therapeutic interventions to refine overall asthma management strategies. Over recent decades, a substantial decline in physical activity levels has resulted in diminished aerobic capacity within the population. This trend has concurrently been observed alongside a dramatic escalation in the prevalence of both asthma and obesity [30]. Provided that asthma is adequately controlled, regular physical conditioning is exceptionally safe in pediatric populations. Numerous empirical investigations strongly suggest that achieving a higher level of cardiorespiratory fitness correlates with significant improvements in asthma symptomatology, overall disease control, and health-related quality of life [31]. Jing et al. suggested, based on their systematic review and meta-analysis, that physical activity can improve forced vital capacity (FVC), forced expiratory flow between 25% and 75% of FVC, and quality of life in pediatric asthmatic patients. Consequently, they recommended physical activity as a valuable adjunctive therapy for children with asthma, with the caveat that it should be avoided in cases of severe asthma or acute exacerbations [32]. These outcomes support the integration of aerobic training into the therapeutic regimen for managing pediatric asthma, suggesting that regular aerobic activity induces physiological adaptations that lead to improved pulmonary function in affected children [33]. Asthmatic subjects who maintain higher levels of physical activity exhibit augmented cytokine secretion following polyclonal stimulation. This suggests a state of preparedness in circulating immune cells for robust type 1, type 2, and type 17 cytokine release when compared to individuals with low physical activity levels. These findings imply that asthmatic children may derive significant immunological benefit from regular exercise, as the observed heightened cytokine response under stimulated conditions indicates an immune system primed to mount a stronger defense against various infectious challenges [34]. Structured exercise interventions have consistently demonstrated favorable outcomes, providing reliable enhancements in cardiorespiratory fitness, clinical asthma symptoms, and the overall quality of life for patients [31].

Exercise in Pediatric Cystic Fibrosis

Physical activity is now recognized as part of multidisciplinary care for people with cystic fibrosis, and interventions lasting at least six months show moderate evidence of improving physical fitness compared to no intervention [35]. Inspiratory muscle training can further increase maximum inspiratory pressure and improve respiratory muscle endurance without observed adverse effects [36]. Due to the frequent persistence of limited physical fitness, which affects reduced life expectancy, patients with cystic fibrosis should be actively encouraged to engage in regular physical activity, and healthcare systems should implement clear strategies for its management [35,37].

Discussion

This systematic review confirms that MVPA is indispensable for optimal pediatric development, with evidence demonstrating that both the intensity and regularity of exercise are critical determinants of health outcomes, aligning with WHO guidelines emphasizing that more physical activity is better [9,29]. As summarized in Table 1, the physiological benefits are profound, notably improving the cardiometabolic risk profile through clinically significant reductions in blood pressure and central adiposity [2,23], while improving lipid panels (e.g., elevated HDL-C) [20]. Furthermore, MVPA acts as a potent, non-pharmacological strategy against insulin resistance by enhancing tissue sensitivity and modulating key adipokines such as adiponectin [11,14]. Concurrently, engagement in physically demanding activities profoundly influences neurocognitive function by strengthening executive functions and conferring an anxiolytic effect via the regulation of dopaminergic/serotonergic pathways [17,19]. Lastly, the mechanical loading inherent in MVPA is critical for skeletal integrity, maximizing the attainment of PBM and increasing BMD/BMC, thereby acting as a crucial, long-term protective factor against adult fracture incidence [26,28]. These comprehensive, dose-dependent benefits underscore the necessity for an interdisciplinary approach to implement structured and often individualized physical activity protocols for all pediatric patients, especially those with coexisting chronic conditions, such as obesity or type 1 diabetes, to maximize therapeutic gain while mitigating specific risks [4,12].

**Table 1:** Clinical efficacy of moderate-to-vigorous physical activity (MVPA) in modulating metabolic and neuropsychiatric health in the pediatric population.

Health domain	Documented clinical and physiological effects	Required intensity and frequency	References
Cardiometabolic health and endocrinology	Lipid profile improvement: significant increase in high-density lipoprotein-cholesterol and reduction in low-density lipoprotein-cholesterol and triglycerides. Vascular risk reduction: mean reduction in blood pressure (5–8 mmHg) and diminished waist circumference (up to 1.5 cm/30 minutes of MVPA). Improved insulin sensitivity: reduction in fasting glucose, fasting insulin, and homeostatic model assessment for insulin resistance. Adipokine modulation: significant elevation of adiponectin	MVPA sustained for ≥60 minutes/day. Minimum effective dose: ≈900−1,200 metabolic equivalent (MET) minutes per week	[2,4,7,12-14,20,22,23]
Neuropsychiatric and cognitive function	Psychosocial enhancement: elevated subjective well-being, increased self-esteem, superior emotional regulation, and reduced symptomatology for anxiety and depression. Biochemical changes: augmentation of serotonergic and dopaminergic pathway activity, conferring an anxiolytic effect. Cognitive performance: crucial enhancement of neurocognitive outcomes and executive functions, including working memory, attentional focus, inhibitory control, and processing speed	Consistent physical activity interventions: ≥30 minutes of daily physical activity for mental health protection	[15,17-19]
Musculoskeletal system integrity	Skeletal development: profound influence on bone homeostasis, leading to a maximal accrual of peak bone mass. Structural gains: significant increase in bone mineral content and bone mineral density, and enhanced skeletal strength and stability. Fracture risk mitigation: marked reduction in the incidence of fractures during development and later in adulthood	Regular physical activity with mechanical loading (bone-strengthening activities); ≥3 days/week; sustained high-level physical activity	[25-29]
Chronic disease management	Juvenile idiopathic arthritis: clinically effective in alleviating pain intensity, increasing functional capacity, and improving health-related quality of life. Type 1 diabetes: reduction in glycated hemoglobin by ≈0.3%. Improved body composition and cardiorespiratory fitness	Individualized, structured exercise interventions (especially for coexisting endocrine disorders)	[8,11,12]

Future recommendations

Future research must primarily focus on refining the dose-response relationship by establishing the optimal, individualized MVPA *dose* in terms of intensity, frequency, and type required for specific pediatric cohorts, particularly those with existing metabolic disorders such as type 1 diabetes and obesity, to maximize clinical efficacy and patient safety. Concurrently, a crucial next step involves strengthening collaborative healthcare through the establishment of formal, interdisciplinary clinical protocols involving pediatricians, endocrinologists, and physical activity specialists. This partnership is necessary for routinely creating and implementing effective individualized exercise interventions for children with chronic health conditions. Furthermore, public health policy should evolve beyond general activity encouragement to promote consistent, higher intensity (MVPA) engagement specifically. Educational efforts, targeting parents and educators, must emphasize that optimal physical, metabolic, and mental health benefits are strongly intensity-dependent. Finally, to fully quantify the long-term public health impact, longitudinal studies are essential to track the retention of healthy MVPA habits formed during childhood into early adulthood, thereby quantifying their protective effect against the development of chronic adult diseases.

## Conclusions

Regular physical activity, especially MVPA, is crucial for physical, metabolic, and mental health, as well as for the proper development of the musculoskeletal system in the pediatric population. The greatest benefits are observed with regular exercise of at least moderate intensity. Studies show that exercise helps maintain a healthy body weight, improves lipid profile, increases insulin sensitivity, and reduces the risk of metabolic disorders, including type 1 diabetes. Consistent physical activity should be regarded as a clinically valuable, safe, and integrative component of pediatric asthma management, given its documented benefits for pulmonary function, symptom control, and immunological preparedness. Regular exercise also supports mental health, reduces the risk of bone fractures, and supports proper skeletal development. Physical activity interventions can be a particularly important element in the treatment of children with chronic diseases, especially type 1 diabetes, obesity, or JIA. For these groups, effective support requires an interdisciplinary approach and individualized recommendations for sick children. It is important to promote the knowledge that not all physical activity will bring commensurate benefits. Often, the desired health effects will only be visible with higher intensity and regularity of physical exercise.
